# Local Strategy Combined with a Wavelength Selection Method for Multivariate Calibration

**DOI:** 10.3390/s16060827

**Published:** 2016-06-04

**Authors:** Haitao Chang, Lianqing Zhu, Xiaoping Lou, Xiaochen Meng, Yangkuan Guo, Zhongyu Wang

**Affiliations:** 1 School of Instrumentation Science & Opto-Electronics Engineering, Beihang University, Beijing 100191, China; changhaitao2005@126.com (H.C.); mewan@buaa.edu.cn (Z.W.); 2 Beijing Key Laboratory for Optoelectronic Measurement Technology, Beijing Information Science & Technology University, Beijing 100192, China; louxiaoping@bistu.edu.cn (X.L.); mengxc@bistu.edu.cn (X.M.); guoyangkuan@tsinghua.org.cn (Y.G.)

**Keywords:** partial least squares regression, wavelength selection, multivariate calibration, ultraviolet-visible absorbance spectra, local algorithm

## Abstract

One of the essential factors influencing the prediction accuracy of multivariate calibration models is the quality of the calibration data. A local regression strategy, together with a wavelength selection approach, is proposed to build the multivariate calibration models based on partial least squares regression. The local algorithm is applied to create a calibration set of spectra similar to the spectrum of an unknown sample; the synthetic degree of grey relation coefficient is used to evaluate the similarity. A wavelength selection method based on simple-to-use interactive self-modeling mixture analysis minimizes the influence of noisy variables, and the most informative variables of the most similar samples are selected to build the multivariate calibration model based on partial least squares regression. To validate the performance of the proposed method, ultraviolet-visible absorbance spectra of mixed solutions of food coloring analytes in a concentration range of 20–200 µg/mL is measured. Experimental results show that the proposed method can not only enhance the prediction accuracy of the calibration model, but also greatly reduce its complexity.

## 1. Introduction

Multivariate entire-spectrum data analysis is recently becoming a hot topic in analytical chemistry. One of the goals of the multivariate spectral analysis is to construct a calibration model that relates spectral databases to the chemical or physical properties of an analytical sample [1]. In complex samples, it is somewhat difficult to discriminate overlapping peaks [2]. Therefore, multivariate calibration methods, like principal components regression (PCR) [3] and partial least squares regression (PLSR) [4], have been extensively used in multivariate spectral analysis. Especially, PLSR has been proven to be a very powerful multivariate statistical tool for quantitative analysis because of its ability to solve problems, such as collinearity and band overlaps of the spectral data [5]. It has been shown that PLSR with global samples can yield precision prediction models.

With the development of full spectrum regression methods, the choice of the most appropriate calibration data is crucial in order to obtain calibration models with good performances in predicting the new samples [6]. Derived from the different directions of selection, the calibration set selection methods can be categorized into two categories: sample selection and variable selection (the later also called wavelength selection).

In the local strategy, the samples (calibration subset) that are spectrally most similar to the one to be predicted are selected from a database (calibration set), and the calibration model has been built on the calibration subset [7]. However, certain key factors, such as the selection method and optimized number of samples in the calibration subset, must be addressed to ensure that the prediction accuracy of the local strategy is significantly better than the one achieved by PLSR with global samples. Various local algorithms based on spectral similarity, including Euclidean distance [8], Mahalanobis distance [9], and spectral angle mapper [10], have been proposed and successfully applied to multivariate calibration. Unfortunately, how to construct the similarity criterion continues to be an unsolved problem.

Wavelength selection in multivariate spectral analysis is an important factor in creating robust models. In some spectral regions, so-called noisy variables may contain information from other analytes, non-modeled interferences, background variations, and interactions that degrade the model accuracy [11]. The benefits of a suitable wavelength selection include not only the stability of the model to the collinearity in the multivariate spectra, but also the interpretability of the relationship between the model and sample compositions [12]. Several approaches can be applied in selecting the most informative variables for multivariate calibration, like genetic algorithms (GA) [13,14], uninformative variable elimination (UVE) [15], moving window PLS (MWPLS) [16], and the successive projections algorithm (SPA) [17]. Generally, the GA, MWPLS, and SPA methods need cross-validation to determine the most appropriate number of selected variables. Unfortunately, the cross-validation is time consuming and slows down the prediction speed of the model. Furthermore, the artificial random variables of UVE and random initial population of GA lead to different results of different runs.

In this paper, PLSR, combined with a local strategy and wavelength selection method, is proposed for the construction of multivariate calibration model. A local algorithm is developed based on the synthetic degree of grey relation coefficient (S-GRC) between the spectrum of the sample to be predicted and each of the spectra in the calibration set. Samples exhibiting the highest correlation are selected to constitute calibration subsets. The root mean square error of prediction (RMSEP) in cross-validation (denoted by RMSECV) during the leave-one-out (LOO) procedure is employed to determine the optimized number of the samples in the calibration subset. The simple-to-use interactive self-modeling mixture analysis (SIMPLISMA) method has been employed to analyze each calibration subset. Pure variables (e.g., wavelengths), characterizing the highest relative standard deviation spectrum values, are selected to build the calibration model by using PLSR. A global model (PLSR based on global samples) with entire spectra, a global model with different wavelength selection methods (such as GA, UVE, MWPLS, SPA), and a local model with different similarity criterions (such as Euclidean distance, Mahalanobis distance, and spectral angle mapper), are also established for comparison. The main innovations in this paper are as follows: (1) S-GRC has been firstly applied as the similarity criterion for local strategy; and (2) considering both improvement of prediction performance and calculation time, SIMPLISMA has been employed as the variable selection method, and the combination of the two methods has also not been found in pervious papers.

## 2. Materials and Methods

### 2.1. Experimental Setup

Visible spectra were measured using a WGS-8 ultraviolet-visible (UV-VIS) spectrophotometer from Tianjin GangDong Science and Technology Development Company, (Tianjin, China). A microcomputer (Lenovo) with an Intel Core 2 processor was used for all the calculations. The analysis was performed using MATLAB software version R2010b. The PLS model and Wilcoxon matched-pairs signed-ranks test were performed with MATLAB function by Stats toolbox. The SIMPLISMA, S-GRC, UVE, MWPLS, SPA, and GA were implemented using in-house software. Reagents of amaranth, carmine, tartrazine, and sunset yellow FCF with a 95% purity were purchased from Standard Material Center (Beijing, China). Doubly-distilled and deionized water was used.

A total of 97 working solutions containing various ratios of amaranth, carmine, tartrazine, and sunset yellow FCF were prepared by appropriate dilution (concentrations of 0–200 µg/mL) with distilled water. The spectra were collected over wavelengths of 407–605 nm at 1-nm intervals using a cuvette with a path length of 1 cm and referenced to an air background; which result in 198 variables. Here, the wavelength range 407–605 nm was considered throughout this paper because the absorbance of all food coloring samples are absent after 605 nm and absorbance of the amaranth and carmine samples are convergent below 407 nm. Each recorded spectrum was the average of 10 successive scans. The 97 samples were then randomly divided into a calibration set (70 samples) and a prediction set (27 samples). It should be noted that to obtain stable and reliable prediction results, the calibration set must be uniformly distributed throughout the sample space.

### 2.2. Data Pre-Processing

**Figure 1:**
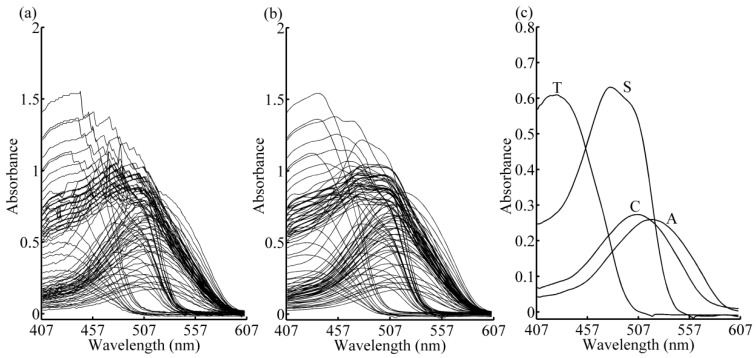
Raw absorption spectra (**a**), filtered spectra with Savitzky-Golay smoothing (**b**), and pure component spectra (**c**) of food coloring samples. A: amaranth; C: carmine; T: tartrazine; S: sunset yellow FCF.

In practical multivariate analysis, a proper pretreatment of the spectral data is necessary. In this paper, the Savitzky-Golay (SG) smoothing was applied to remove the noise and distortion in the original spectra. The parameters have been set as follows: degree of polynomial *p* = 2, number of smoothing points 2*l* + 1 = 21. The original absorption spectra and smoothed spectra of the 97 food coloring samples are shown in Figure 1a,b. The original spectra displayed in Figure 1a, which can be observed that the spectra are often distorted, especially with high concentrations near the maximum absorption positions of the samples, leading to a nonlinearity in the spectra. Figure 1b displays the smoothed spectra with SG smoothing method. It is obvious that the smoothed spectra maintain the important features of the original spectra such as maximum absorption positions and overall shape by comparison with Figure 1a. Although the SG smoothing method produces a superior estimate for spectra data, there is a clear overlapping of the spectra and the datasets include nonlinearity and irrelevant variables. Figure 1c shows the absorption spectra of 60 µg/mL aqueous solutions with single components, such as amaranth, carmine, tartrazine, and sunset yellow FCF. As can be seen, the spectra of amaranth and carmine overlap, and bands in the sunset yellow FCF spectrum overlap with the absorbing regions of the other analytes. Thus, straightforward UV-VIS absorbance measurements are not able to distinguish these compounds; therefore, multivariate calibration is a suitable choice for overcoming this problem.

### 2.3. Local Strategy

Local strategy is based on the selection of a calibration subset from a spectral database for each unknown sample. This method is especially suitable for the spectra which have grouping information according to different composition. Each unknown sample requires the development of a specific model with a new subset of samples that are spectrally similar. The selection of a calibration subset is a critical step that considerably affects the precision and accuracy of the subsequent calibration. The similarity between each predicted sample and samples in calibration set has been computed using the S-GRC, and the calibration subset is selected on the basis of the higher S-GRC. This calculation step is described in detail in the following paragraph. To achieve the best prediction performance using the local strategy, the number of samples in calibration subset for each prediction sample needs to be evaluated. In this study, LOO cross-validation is applied, and RMSECV is calculated to determine the number of samples in the calibration subset. The optimal model always shows the lowest RMSECV.

Grey system theory [18] is a useful mathematical tool for analyzing systems when a limited amount of information is available. It has been widely applied in various fields [19,20]. Grey relation analysis (GRA) is one tool of grey system theory used for determining whether sequences are closely related [21,22]. Here we propose the S-GRC to fully evaluate the similarity between absorption spectra of samples by analyzing the absolute deviation and change rates of the sequences.

For computing the S-GRC between reference sequence *X_i_* and sequence *X_j_* the following equation is used:

(1) $$\rho (X_{i},X_{j})=\theta \varepsilon_{ij}+(1-\theta )\cdot \gamma_{ij}$$

where the sequences *X_i_* and *X_j_* are n × 1 vectors from prediction set and calibration set respectively, n is the number of wavelengths, $i=1,2,\cdots ,m_{p}$, $j=1,2,\cdots ,m_{c}$, $m_{p}$ and $m_{c}$ are number of samples in prediction set and calibration set, respectively, $\varepsilon_{ij}$ is the absolute degree of GRC, $\gamma_{ij}$ is the relative degree of GRC, and θ∈[0,1] is the weight of the change rates. In this paper, the relative degree of GRC is focused on the geometrical difference between spectra sequences and the effect of based bias between different spectrums can be eliminated. Therefore, it is better than the absolute degree of GRC in discrimination of overlapping spectra. Therefore, the weight value θ is set to be 0.2. The $\varepsilon_{ij}$ represents difference of sequences in absolute deviation, which is given by:

(2) $$\varepsilon_{ij}=\frac{1+\left|s_{i}\right|+\left|s_{j}\right|}{1+\left|s_{i}\right|+\left|s_{j}\right|+\left|s_{i}-s_{j}\right|}$$

where $\left|s_{i}\right|$, $\left|s_{j}\right|$ and $\left|s_{i}-s_{j}\right|$ are calculated as follows:

(3) $$\left|s_{i}\right|=\left|\sum_{k=1}^{n-1}x_{i}(k)+0.5x_{i}(n)\right|$$

(4) $$\left|s_{j}\right|=\left|\sum_{k=1}^{n-1}x_{j}(k)+0.5x_{j}(n)\right|$$

(5) $$\left|s_{i}-s_{j}\right|=\left|\sum_{k=1}^{n-1}\left(x_{i}(k)-x_{j}(k)\right)+0.5\left(x_{i}(n)-x_{j}(n)\right)\right|$$

where $x_{i}(k)\in X_{i}$ and $x_{j}(k)\in X_{j}$. The $\gamma_{ij}$ describes the difference in geometry between sequences, which is calculated by:

(6) $$\gamma_{ij}=\frac{1}{n}\sum_{k=1}^{n}\gamma (x_{j}(k),x_{i}(k))$$

where $\gamma (x_{j}(k),x_{i}(k))$ is given by:

(7) $$\gamma (x_{j}(k),x_{i}(k))=\frac{\underset{l=1,\cdots ,m_{c}}{\min}\left|x_{l}(k)-x_{i}(k)\right|+\xi \underset{l=1,\cdots ,m_{c}}{\max}\left|x_{l}(k)-x_{i}(k)\right|}{\left|x_{j}(k)-x_{i}(k)\right|+\xi \underset{l=1,\cdots ,m_{c}}{\max}\left|x_{l}(k)-x_{i}(k)\right|}$$

and ξ∈[0,1] is the distinguishing coefficient. According to [22] the value is generally set at ξ=0.5. Here, $\underset{l=1,\cdots ,m_{c}}{\min}\left|x_{l}(k)-x_{i}(k)\right|$ and $\underset{l=1,\cdots ,m_{c}}{\max}\left|x_{l}(k)-x_{i}(k)\right|$ are, respectively, the minimum value and maximum value of the $1\;\times \;m_{c}$ deviation vector, where *l* is from 1 to *m*_c_. The symbols used in the calculation process of S-GRC are summarized in Table 1.

### 2.4. Wavelength Selection

For each unknown sample, a new calibration model has been employed with local strategy. Compared with global method, the computation load of local strategy will be greatly increased. The traditional wavelength selection methods is complex when they are used in a local strategy. Thus, considering both effectiveness and time consumption, the SIMPLISMA can be used as the wavelength selection method for the local model in this paper.

The goal of wavelength selection is to eliminate noisy variables and to improve the prediction performance. Wavelength selection based on SIMPLISMA can provide the analytical wavelengths with a high signal to noise ratio (called pure variables) for the prediction model and the influence of the variables that are irrelevant to the studied properties can be eliminated. The pure variable is one that contains intensity contributions from only one component in the mixture [23], and SIMPLISMA assumes that every component in the mixture under study has a pure variable (e.g., a wave number) with a finite intensity for the particular component and zero intensity for all other components in the mixture [24]. Since pure-variable intensities are directly proportional to the concentrations of the associated components, a calibration model constructed with pure wavelengths provides better results than that constructed with the entire spectrum. The number of the selected variables is a critical parameter, which decides the stability and accuracy of the model. When the number of selected variables is too small, the robustness and accuracy of the model may be affected due to the loss of useful informative variables. On the other hand, when more variables are used, uninformative variables may be contained in the model and cause its performance to be weak. Here, in order to generate a rapid process of wavelength selection, the determining coefficient function without cross-validation, defined in Section 2.5, has been used to determine the proper number of the selected wavelengths.

### 2.5. Simple-to-Use Interactive Self-Modeling Mixture Analysis

In the SIMPLISMA method [25], the pure variable is determined by the standard deviation divided by the sum of the mean and a constant:

(8) $$p_{ij}=w_{ij}\frac{{\bar{\sigma }}_{i}}{{\bar{\mu }}_{i}+\alpha }$$

where $p_{ij}$ represents the purity values of the selected variable (*i* = 1, 2, …, *n*; *j* is the number of pure variables, and *j* = 1, 2, …, *r*; r is the number of components). All of the $p_{ij}$ values are plotted in the form of a spectrum, the so-called purity spectrum, and the wavenumber of the highest intensity represents the *j*th pure variable. α is the value of noise. This noise level is typically l–5% of the maximum mean, and here, we use a value of 1%. ${\bar{\mu }}_{i}$ is the mean and ${\bar{\sigma }}_{i}$ is the standard deviation of the *i*th column vector of the original spectral data matrix $D_{m\times n}$, *m* is the number of samples.

(9) $${\bar{\mu }}_{i}=\frac{1}{m}\sum_{j=1}^{m}d_{ij}$$

(10) $${\bar{\sigma }}_{i}=\sqrt{\frac{1}{m}\sum_{j}^{m}{\left(d_{ij}-{\bar{\mu }}_{i}\right)}^{2}}$$

The weight factor $w_{ij}$ is a determinant-based function used to remove all the contributions correlating with previously pure variables:

(11) $$w_{i,j}=\left|\begin{array}{cccc} C_{i,i} & C_{i,p_{1}} & \cdots & C_{i,p_{j-1}}\\ C_{p_{1},i} & C_{p_{1},p_{1}} & \cdots & C_{p_{1},p_{j-1}}\\ \cdots & \cdots & \cdots & \cdots \\ C_{p_{j-1},i} & C_{p_{j-1},p_{1}} & \cdots & C_{p_{j-1},p_{j-1}} \end{array}\right|$$

Note that $w_{i,1}=1$ for *j* = 1. Here, $p_{j-1}$ is the wave number of the *(j −* 1*)*th purity variable, and *C* is the correlation around the origin matrix, which is given by:

(12) $$C=(1/m)D(\lambda )D{\left(\lambda \right)}^{T}$$

where D(λ) is the original data matrix *D* scaled by the length λ ($\lambda_{i}=\sqrt{\mu_{i}^{2}+{\left(\sigma_{i}+\alpha \right)}^{2}}$; $d{\left(\lambda \right)}_{k,i}=d_{k,i}/\lambda_{i}$, *k* = 1, 2, …, *m*; *i* = 1, 2, …, *n*). The standard deviation spectrum $s_{i,j}$ that has the closest relationship with the original data is described by:

(13) $$s_{i,j}={\bar{\sigma }}_{i}w_{i,j}$$

SIMPLISMA will continue to search for pure variables until the maximum number of components is reached. The number of pure variables is determined on the basis of determining coefficient defined as:

(14) $$R_{j}=R_{sj}/R_{s(j+1)}$$

where *R_j_* is the determining coefficient of the *j*th pure variable, and $R_{sj}$ is the relative total intensity of the standard deviation spectra of the *j*th pure variable, which is given by:

(15) $$R_{sj}=\sum_{i=1}^{n}s_{i,j}/\sum_{i=1}^{n}s_{i,1}$$

If the *j* pure variables are representative of the entire mixture system, then all the other variables in the dataset will be linear combinations of these *j* pure variables, resulting in $R_{s(j+1)}$ value of nearly zero. Since the value of $R_{s(j+1)}$ become close to 0, the value for determining coefficient *R_j_* will be relatively high after determining the proper number of pure variables. Additional details regarding this method can be found in [23,25].

### 2.6. Model Evaluation

The RMSEP of the prediction set was used to evaluate the accuracy of our models, with the RMSEP calculated as:

(16) $$RMSEP=\sqrt{\frac{\sum_{j=1}^{m_{p}}{\left(y_{j}-{\hat{y}}_{j}\right)}^{2}}{m_{p}}}$$

where ${\hat{y}}_{j}$ and $y_{j}$ are the predicted and reference concentrations of the *j*th sample, and $m_{p}$ is the number of samples in the prediction set.

Statistical analysis was performed using the Wilcoxon matched-pairs signed-ranks test between reference and predicted concentrations of different methods [26]. In this paper, an ”exact” test was used and two-tailed p values were calculated. Differences were considered statistically significant at p < 0.05.

For the local strategy, RMSECV of the LOO procedure was used to optimize the number of samples in calibration subset, which is given by:

(17) $$RMSECV=\sqrt{\frac{\sum_{j=1}^{N}{\left(y_{j}-{\hat{y}}_{\left(j\right)}\right)}^{2}}{N}}$$

where $y_{j}$ is the concentration of the *j*th sample in calibration set, ${\hat{y}}_{\left(j\right)}$ is the predicted values in cross-validation without the *j*th sample, and N is the number of samples in calibration subset, $N\leq m_{c}$.

In order to select the optimum PLS factors, leave-one-out cross-validation (LOOCV) was used. Here, LOOCV works by temporarily extracting one sample from calibration set, and then predicting the selected sample by the remaining ones. Since the concentrations of the samples in calibration set are known, the prediction errors can be calculated. In this situation, the LOOCV process is repeated as many times as there are samples in calibration set. The squared prediction errors are summed and expressed as the prediction residual error sum of squares (PRESS), which is calculated by:

(18) $$PRESS_{k}=\sum_{j=1}^{m_{c}}{\left(y_{j}-{\hat{y}}_{\left(j\right)}\right)}^{2}$$

where $m_{c}$ is the number of calibration samples; $y_{j}$ the reference concentration for *j*th sample and ${\hat{y}}_{\left(j\right)}$ represents the estimated concentration. Resembling the F test, the determination of the PLS factors is mathematically defined by computing the ratio between two successive values of PRESS. If $PRESS_{k}/PRESS_{k-1}$ exceeds 1, use (*k −* 1) PLS factor in the model.

As comparison, the local algorithm based on the Mahalanobis distance has also been calculated, which is given by:

(19) $$MD_{j}^{2}=\left(T_{j}-T_{pred}\right)COV^{-1}{\left(T_{j}-T_{pred}\right)}^{-1}$$

where $MD_{j}$ is the Mahalanobis distance *j*th sample and predicted sample, $T_{j}$ and $T_{pred}$ are the scores of *j*th sample and predicted sample, and COV is the covariance matrix of the scores matrix of the calibration set.

## 3. Results and Discussion

### 3.1. Calibration Subset Selection

A key factor influencing the predictions accuracy is the choice of an adequate size and distribution of the calibration subset. In this study, the proper number of samples in the calibration subset that returns the minimum RMSECV of the LOO procedure was identified. Although the calibration model may be not the best in this method, it was robust and realized acceptable accuracy. The calibration subset selection steps are as follows:

1. Sorting calibration set $A_{m\times n}$ in the descending order of S-GRC values. Here, *m* = 70 and *n* = 198;
2. Selecting the former *N_sub_* samples of calibration set that compose calibration subset. Here, *N_sub_* = 3 or 4 or 5;
3. Applying the regression method, PLSR, on the absorbance data in the calibration subset using the LOOCV method, calculating the RMSECV; and
4. *N_sub_* = *N_sub_* + 1, repeating the step (2) and (3) until *N_sub_* > 70.

**Figure 2:**
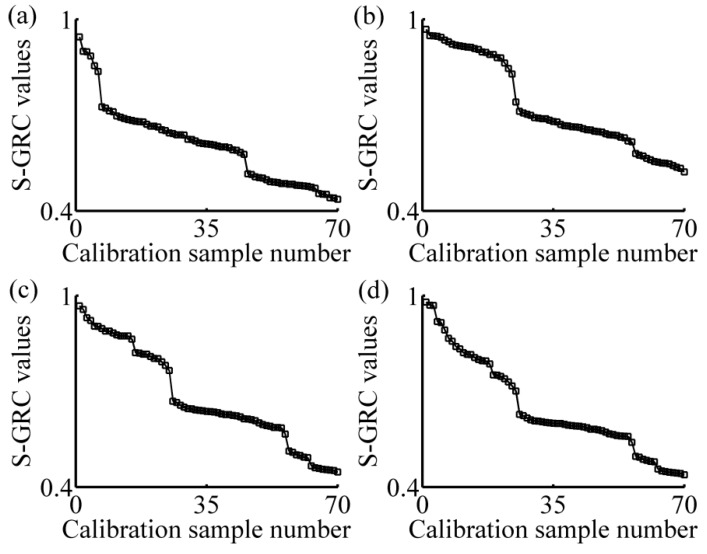
Variation of the S-GRC with the number of calibration samples. (**a**) two-component sample 1 (tartrazine: 60 μg/mL, sunset yellow FCF: 50 μg/mL); (**b**) four-component sample (amaranth: 50 μg/mL, carmine: 60 μg/mL, tartrazine: 40 μg/mL, sunset yellow FCF: 30 μg/mL); (**c**) two-component sample 2 (amaranth: 100 μg/mL, carmine: 50 μg/mL); (**d**) one-component sample (amaranth: 160 μg/mL). SGRC: synthetic degree of grey relation coefficient.

Figure 2a–d show the variation of S-GRC with the number of calibration samples. Since the samples in the calibration set have been reordered in descending order of S-GRC, all S-GRC curves have a descending trend. It can be seen that, at the beginning, the S-GRC values are comparatively large. Along with the increase of the calibration samples number, the descending trend of S-GRC is from sharp to slow. Sudden change in S-GRC curve values resulted from the changes in composition of similar samples. This indicates that the S-GRC curve showed partial sample grouping according to different composition.

**Figure 3:**
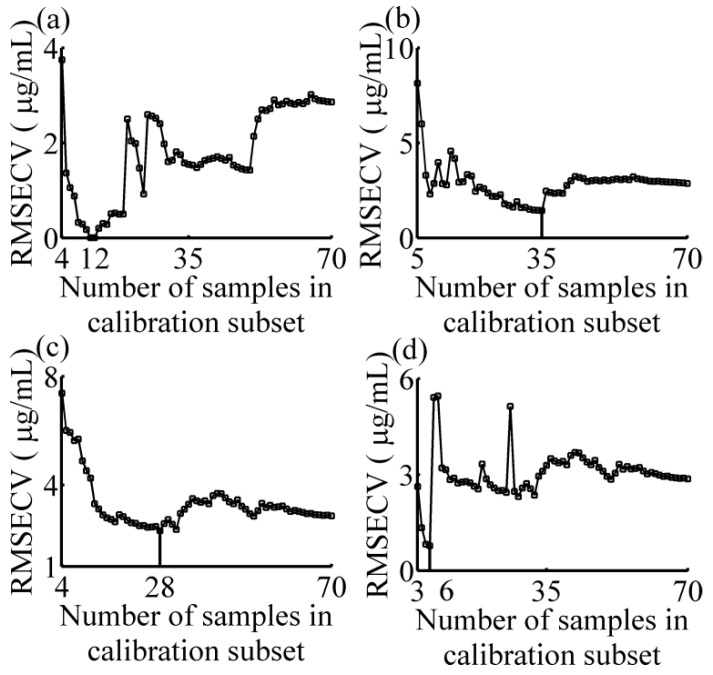
RMSECV of the partial least squares model as a function of the number of samples in the calibration subset. (**a**) two-component sample 1 (tartrazine: 60 μg/mL, sunset yellow FCF: 50 μg/mL); (**b**) four-component sample (amaranth: 50 μg/mL, carmine: 60 μg/mL, tartrazine: 40 μg/mL, sunset yellow FCF: 30 μg/mL); (**c**) two-component sample 2 (amaranth: 100 μg/mL, carmine: 50 μg/mL); (**d**) one-component sample (amaranth: 160 μg/mL). RMSECV: root mean square error of the cross validation.

**Figure 4:**
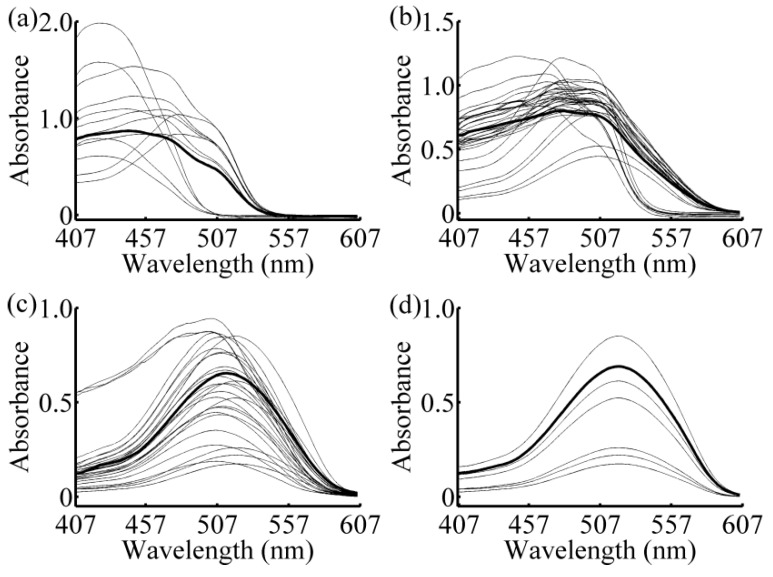
Absorption spectra of calibration subset. (**a**) two-component sample 1 (tartrazine: 60 μg/mL, sunset yellow FCF: 50 μg/mL); (**b**) four-component sample (amaranth: 50 μg/mL, carmine: 60 μg/mL, tartrazine: 40 μg/mL, sunset yellow FCF: 30 μg/mL); (**c**) two-component sample 2 (amaranth: 100 μg/mL, carmine: 50 μg/mL); and (**d**) one-component sample (amaranth: 160 μg/mL).

The variations of the RMSECV with the number of samples in calibration subset for four different predicted samples are shown in Figure 3a–d. With the increase of the samples in calibration subset, the general trend of all curves are firstly decreased (except one-component sample), and then increased, and finally stable with little fluctuation. This indicates that with a smaller number of samples, similar samples cannot be completely included, so the model quality is not good. On the contrary, if the number of samples in calibration subset is too large, dissimilar samples may be contained in the model and make the RMSECV remain at a comparatively large value. Here, when the numbers of samples in calibration subset are 12, 35, 28, and 6, respectively, the lowest values of RMSECV are obtained. Therefore, the numbers of samples in calibration subsets are set 12, 35, 28, and 6 for further study. The spectra of calibration subset for different samples are shown in Figure 4. The thick curves are the absorbance spectrum of predicted samples, and the fine curves are the absorbance spectra of the calibration subset samples. It is obvious that not only the samples with the smaller absolute deviation, but also samples with similar rates of change have been selected in the calibration subset. The concentrations of each sample in calibration subset are list in Table 2. The S-GRC between predicted sample and the samples in calibration subset are also presented.

### 3.2. Wavelength Selection

**Figure 5:**
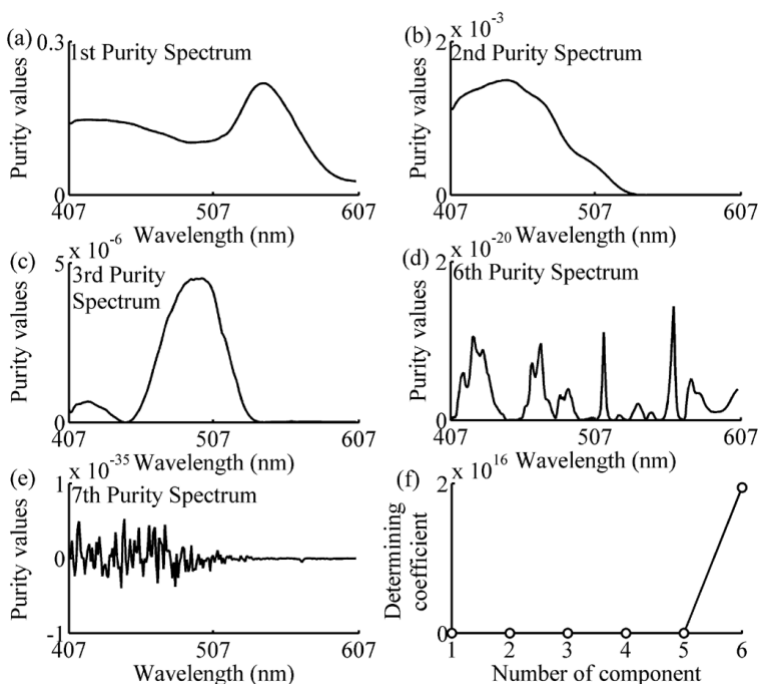
Purity spectra and determining coefficient during the processing of two-component sample’s calibration subset spectra. (**a**) 1st purity spectrum; (**b**) 2nd purity spectrum; (**c**) 3rd purity spectrum; (**d**) 6th purity spectrum; (**e**) 7th purity spectrum; (**f**) determining coefficient. The components are tartrazine (60 μg/mL) and sunset yellow FCF (50 μg/mL).

**Figure 6:**
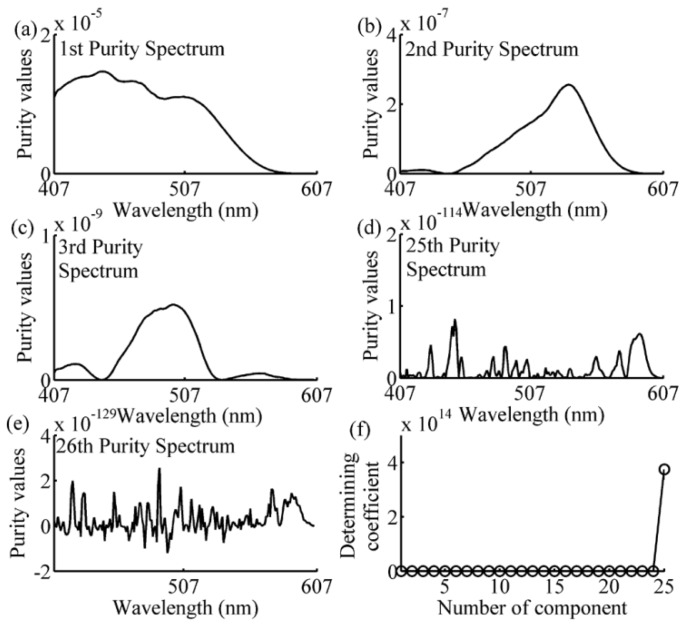
Purity spectra and determining coefficient during the processing of four-component sample’s calibration subset spectra. (**a**) 1st purity spectrum; (**b**) 2nd purity spectrum; (**c**) 3rd purity spectrum; (**d**) 25th purity spectrum; (**e**) 26th purity spectrum; (**f**) determining coefficient. The components are amaranth (50 μg/mL), carmine (60 μg/mL), tartrazine (40 μg/mL), and sunset yellow FCF (30 μg/mL).

**Figure 7:**
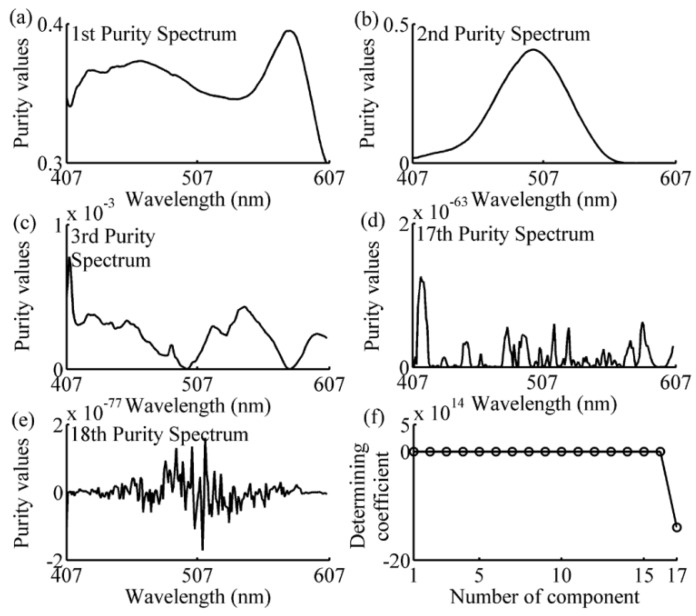
Purity spectra and determining coefficient during the processing of two-component sample’s calibration subset spectra. (**a**) 1st purity spectrum; (**b**) 2nd purity spectrum; (**c**) 3rd purity spectrum; (**d**) 17th purity spectrum; (**e**) 18th purity spectrum; (**f**) determining coefficient. The components are amaranth (100 μg/mL) and carmine (50 μg/mL).

**Figure 8:**
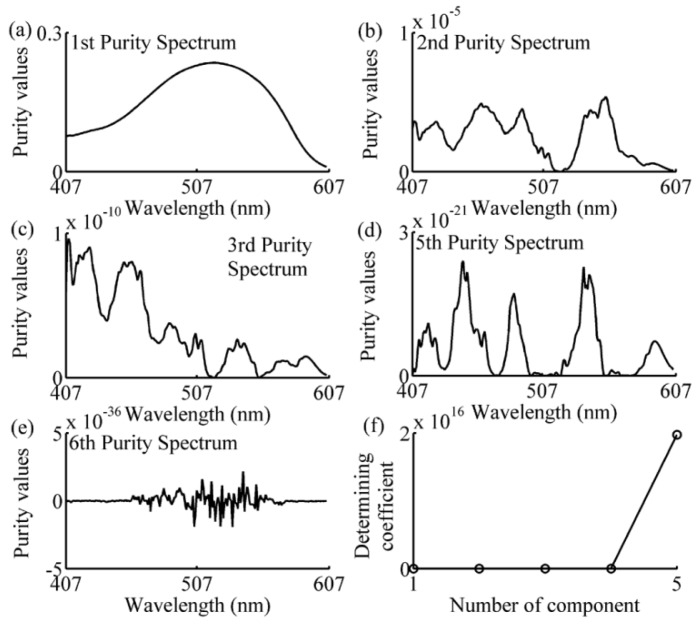
Purity spectra and determining coefficient during the processing of one-component sample’s calibration subset spectra. (**a**) 1st purity spectrum; (**b**) 2nd purity spectrum; (**c**) 3rd purity spectrum; (**d**) 5th purity spectrum; (**e**) 6th purity spectrum; (**f**) determining coefficient. The component is amaranth (160 μg/mL).

The spectral data in the calibration subset of each prediction sample was analyzed using the SIMPLISMA approach with a noise level α of 1%, as can be seen in Equations (8)–(13). The number of the selected wavelengths has been determined by the coefficient defined in Equation (14). The following examples of one-component, two-component 1–2, and four-component samples, illustrated the process of wavelength selection are shown in Figure 5, Figure 6, Figure 7 and Figure 8. As can be seen in Figure 5, the variables with a relatively high intensity (between 517–572 nm, in Figure 5a) will be relatively pure, and the variable with the highest intensity (at 542 nm, shown in Figure 5a) is the first pure variable. By contrast, a variable with a low intensity (between 407–507 nm, in Figure 5a) will have contributions from several components. After eliminating the effect of the first pure variable by using Equations (11) and (12), the second purity spectrum is shown in Figure 5b. The second purity spectrum results in the selection of the next pure variable, *i.e.*, 447 nm, which is accepted as the second pure variable. Following this treatment, the other pure variables are selected, until the number of the pure variables equals 7. As shown in Figure 5e, the seventh purity spectrum has an odd shape and low intensities nearly zero. Such erratic behavior is a strong indication that spectrum consists of only noise. This is also confirmed by determining coefficient curve in Figure 5f. As shown in Figure 5f, there is a sudden change of determining coefficient when *j* = 6. This indicates that the relative total intensity of the standard deviation spectra of the seventh pure variable defined in Equation (15) is nearly zero and the seventh purity spectrum does not contain any useful information except the noise. Thus, the number of the selected wavelength is 6, and the SIMPLISMA process ends at the number of pure variables equaling to 7. With the same method, the purity spectra and determining coefficient during the processing of calibration subset spectra for four-component sample, two-component sample 2 and one-component sample, are shown in Figure 6, Figure 7 and Figure 8, respectively. As can be seen, the number of selected wavelength for four-component sample (amaranth (50 μg/mL), carmine (60 μg/mL), tartrazine (40 μg/mL), and sunset yellow FCF (30 μg/mL)), two component sample (amaranth (100 μg/mL) and carmine (50 μg/mL)) and one component sample (amaranth (160 μg/mL)) are 25, 17, and 5, respectively.

**Figure 9:**
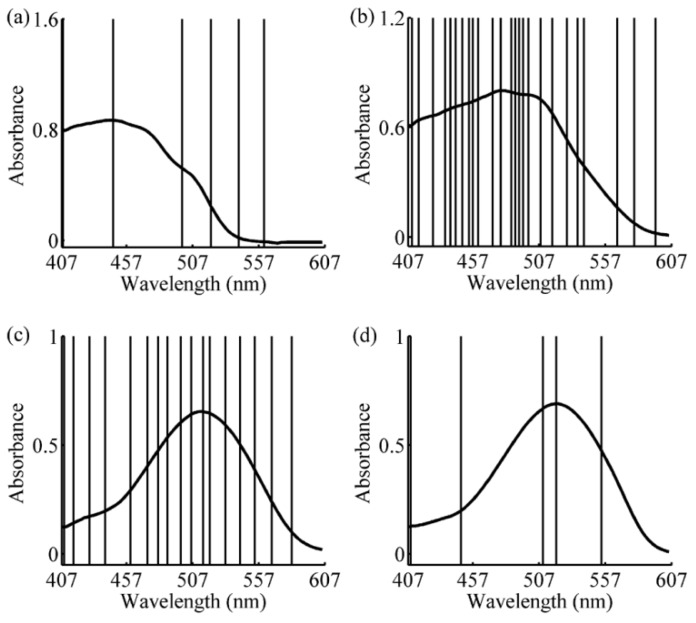
Wavelengths selected by simple-to-use interactive self-modeling mixture analysis. (**a**) Two-component sample 1 (tartrazine: 60 μg/mL, sunset yellow FCF: 50 μg/mL); (**b**) four-component sample (amaranth: 50 μg/mL, carmine: 60 μg/mL, tartrazine: 40 μg/mL, sunset yellow FCF: 30 μg/mL); (**c**) two-component sample 2 (amaranth: 100 μg/mL, carmine: 50 μg/mL); and (**d**) one-component sample (amaranth: 160 μg/mL).

As shown in Figure 9a,d, according to the determining coefficients 6, 25, 17, and 5 wavelengths were selected, respectively, for the specific examples. It is clear that the number of selected wavelengths (indicated by the vertical lines) in Figure 9b,c are greater than that in Figure 9a,d. This is due to interference between the components with overlapping spectra in the four-component sample (shown in Figure 9b) and two-component sample 2 (shown in Figure 9c).

The final size of the calibration subset for each unknown sample in prediction set has been shown in Table 3. It can be seen that the one-component samples and the two-component samples, like the first, 6–10, and 16–21 prediction samples, have a small number of samples and wavelengths in the calibration subset (less than 12). Two-component samples, like 15th, 22nd, and 23rd prediction samples, have relatively larger calibration subset sizes; this is because there is a clear overlapping of the spectra of the two components. For the four-component samples, the sizes of calibration subsets are usually more than 20, to obtain adequate similar samples for building a prediction model.

### 3.3. Comparative Performance of the Various Methods

The RMSEPs of the prediction set (27 samples) and computational time are calculated by using the proposed method and listed in Table 4. As a comparison, the RMSEPs and computational time obtained by a global model with entire spectra, a global model combined with GA, UVE, MWPLSR, SPA wavelength selection methods, and a local model with Euclidean distance, Mahalanobis distance, and spectral angle mapper similarity criterions with the same calibration and prediction set are also listed in the table. It should be noted that after selecting a calibration set or subset with one of the aforementioned methods, calibration equations were calculated using PLSR and LOOCV was used to select the optimum number of PLS latent variables. The results of different runs with UVE can be different, so the time and RMSEP of UVE are the averages of 10 runs.

As shown in Table 4, it is clear that all local strategies with different similarity criteria can improve the prediction performance which produced the smaller RMSEP of each component compared with global model with entire spectra. Especially, a local strategy with a S-GRC similarity criterion can provide a relative RMSEP improvement of amaranth, carmine, tartrazine, and sunset yellow FCF greater than 60%, 57%, 12%, and 40%, respectively, than obtained by a global model with entire spectra. In addition, each query sample requires the development of a specific model with a new subset of samples, so all local strategies need long computational times, more than 120 s. As expected, the lower RMSEP has also been obtained with wavelength selection methods. However, the GA, MWPLS, and SPA methods need cross-validation to determine the proper number of selected variables, so the calculation time is obviously longer than SIMPLISMA and UVE methods. Considering both prediction performance and calculation time, the SIMPLISMA and UVE have been chosen as the wavelength methods for calibration subsets selected by the local strategy in this paper. Comparison of the RMSEPs obtained by these two methods with the same local strategy shows that the SIMPLISMA produced a better prediction with smaller RMSEPs. Although the RMSEP of carmine obtained by the local model combined with SIMPLISMA is slightly larger than that by UVE, there is no significant difference between 1.76 µg/mL and 1.73 µg/mL. In addition, the final size of selected calibration set obtained by local strategy based on S-GRC combined with SIMPLISMA wavelength selection is [4,38] × [3,25], which is considerably less than that with UVE. Such results also indicate the proposed method is more effective. It should be noted that [4,38] × [3,25] represents the number of samples in all selected calibration sets, between 4 and 38, and the number of selected variables in these sets is between 3 and 25.

Statistical analysis was also performed using the Wilcoxon matched-pairs signed-ranks test. The p-values between reference and predicted concentrations with different methods were listed in Table 5. As can be seen in Table 5, no statistical differences were found between reference and predicted concentrations with different methods for each component compared with Wilcoxon tests (p > 0.05). This means it is hard to evaluate the prediction performance of different models based merely on Wilcoxon tests. Therefore, standard error of prediction residual error was further calculated. As shown in Table 4 and Table 5, the standard errors of prediction residual error and RMSEP are almost equal, and with a similar variation tendency. This proves, yet again, that both local strategy and wavelength selection approaches can improve the prediction performance of multivariate calibration models, especially the local strategy of S-GRC, wavelength selection based on SIMPLISMA, and a combination of both.

## 4. Conclusions

Throughout this paper, we proposed to conduct a local strategy based on the similarity criterion named as S-GRC. RMSECV has been applied to determine the number of spectrally-similar samples for each unknown sample. Wavelength selection has been performed according to the order ranking of pure variables by SIMPLISMA. In order to generate rapid predictions, a determining coefficient has been used to ascertain the proper number of selected variables without cross-validation. For comparison, a global model with an entire spectra, a global model with different wavelength selection methods (GA, UVE, MWPLS, SPA), and a local model with different similarity criteria (Euclidean distance, Mahalanobis distance, and spectral angle mapper) were also developed. With ultraviolet-visible spectral data of food coloring analytes, it has been proved that an optimized calibration subset can be selected by the proposed methods for building a high-performance prediction model in reasonable time frames. In conclusion, the local strategy based on S-GRC, combined with the SIMPLISMA wavelength selection method, was recommended to build a robust model for multivariate calibration, especially to resolve spectra with partial overlaps.

## Figures and Tables

**Table 1:** The summary of symbols used in the calculation process of S-GRC.

Symbol	Representation
θ=0.2	weight of the rates of change
ξ=0.5	distinguishing coefficient
$\rho (X_{i},X_{j})$	synthetic degree of GRC between sequences *X*_i_ and *X*_j_
$\varepsilon_{ij}$	absolute degree of GRC between sequences *X*_i_ and *X*_j_
$\gamma_{ij}$	relative degree of GRC between sequences *X*_i_ and *X*_j_
n	the number of wavelengths
$m_{p}$	number of samples in prediction set
$m_{c}$	number of samples in calibration set

S-GRC: Synthetic degree of grey relation coefficient.

**Table 2:** The concentrations of each sample in calibration subset.

Samples	No.	SGRC	S1 (μg/mL)	SGRC	S2 (μg/mL)	SGRC	S3 (μg/mL)	SGRC	S4 (μg/mL)
Samples of Calibration subset	1	0.945	0-0-80-60	0.969	60-60-40-30	0.968	80-60-0-0	0.980	140-0-0-0
	2	0.900	0-0-100-100	0.949	50-80-50-30	0.957	100-60-0-0	0.970	200-0-0-0
	3	0.899	0-0-80-80	0.949	60-80-50-30	0.930	100-80-0-0	0.970	120-0-0-0
	4	0.885	0-0-50-60	0.947	60-80-50-40	0.922	80-80-0-0	0.920	50-0-0-0
	5	0.855	0-0-60-80	0.944	60-60-50-40	0.904	60-50-0-0	0.920	60-0-0-0
	6	0.837	0-0-50-80	0.935	50-60-40-40	0.904	100-100-0-0	0.892	40-0-0-0
	7	0.726	0-0-80-0	0.930	60-80-40-40	0.900	80-100-0-0	─	─
	…	…	…	…	…	…	…	…	─
	12	0.693	0-0-0-80	0.913	80-60-40-40	0.874	200-0-0-0	─	─
	13	─	─	0.913	80-80-50-30	─	50-80-0-0	─	─
	…	…	…	…	…	…	…	…	…
	28	─	─	0.703	0-180-0-0	0.656	60-80-30-30	─	─
	…	…	…	…	…	…	…	…	…
	35	─	─	0.680	0-0-80-80	─	─	─	─
Predicted sample			0-0-60-50		50-60-40-30		100-50-0-0		160-0-0-0

SGRC: synthetic degree of grey relation coefficient; ─: absence of samples; S1: two-component sample 1 (tartrazine: 60 μg/mL, sunset yellow FCF: 50 μg/mL); S2: four-component sample (amaranth: 50 μg/mL, carmine: 60 μg/mL, tartrazine: 40 μg/mL, sunset yellow FCF: 30 μg/mL); S3: two-component sample 2 (amaranth: 100 μg/mL, carmine: 50 μg/mL); S4: one-component sample (amaranth: 160 μg/mL).

**Table 3:** The final size of the calibration subset for each unknown sample in the prediction set.

Prediction Sample	Number of Samples in Calibration Subset	Number of Selected Wavelengths	Prediction Sample	Number of Samples in Calibration Subset	Number of Selected Wavelengths
1	12	6	15	34	19
2	35	25	16	7	3
3	37	18	17	5	6
4	13	12	18	6	5
5	38	16	19	5	5
6	7	8	20	4	5
7	7	3	21	4	5
8	5	6	22	28	17
9	5	6	23	10	11
10	4	4	24	13	12
11	12	5	25	33	17
12	34	18	26	38	16
13	26	15	27	5	5
14	34	18	-	-	-

**Table 4:** Prediction results obtained using four methods.

Model	Wavelength Selection Approach	Parameters	Size of Calibration Set	Times (s)	RMSEP (µg/mL)			
					A	C	T	S
Global	―	―	70 × 198	0.4	4.12	4.15	1.01	0.99
	SIMPLISMA	*R_j_* = 2 × 10^−5^	70 × 19	0.4	3.71	3.72	0.97	1.08
	GA	*Gen* = 300	70 × 75	3666.6	2.74	3.05	1.14	1.28
	UVE	$Noise\in [0,\;10^{-2}]$	70 × [100,177]	4.2	4.01	3.92	0.99	1.07
	MWPLSR	*Win* = 60	70 × 60	57.1	3.01	2.17	1.34	1.12
	SPA	*M* = 69	70 × 69	98.9	3.76	3.61	1.00	1.03
Local^S^	―	θ = 0.2, ξ = 0.5	[4,38] × 198	123.1	1.63	1.77	0.88	0.59
Local^E^	―	―	[4,34] × 198	145.4	3.14	3.97	0.76	0.80
Local^M^	―	*f_pca_* = 2	[4,32] × 198	152.7	2.15	1.95	0.63	0.81
Local^A^	―	―	[3,40] × 198	156.6	2.99	2.53	0.81	0.77
Local^S^	SIMPLISMA	*R_j_* = 2 × 10^−5^	[4,38] × [3,25]	128.2	1.41	1.76	0.72	0.68
	UVE	$Noise\in [0,\;10^{-2}]$	[4,38] × [65,174]	136.8	1.64	1.73	1.06	0.76

SIMPLISMA = the simple-to-use interactive self-modeling mixture analysis. UVE = uninformative variables elimination. MWPLSR = moving window partial least squares. SPA = successive projections algorithm. GA = genetic algorithm. Local^S^ = local strategy based on synthetic degree of grey relation coefficient. Local^E^ = local strategy based on Euclidean distance. Local^M^ = local strategy based on Mahalanobis. Local^A^ = local strategy based on Angle. *R_j_* is the determining coefficient of the *j*th pure variable. The *Noise* is the added artificial random variable in UVE method whose values are from 0 to 10^−2^. *Win* is the size of moving window when using MWPLSR. *M* is the number of variables with SPA method. According to Res. 17, *M* = min (*m_c_* − 1, *n*) = 69, where $m_{c}$ and *n* are the number of samples and wavelengths in calibration set. *Gen* is the maximum number of generations in GA. θ is the weight of the rates of change. ξ is a distinguishing coefficient. *f_pca_* is the number of principal components. RMSEP = root mean square error of prediction; A: amaranth; C: carmine; T: tartrazine; S: sunset yellow FCF.

**Table 5:** Statistical analysis of prediction values with different methods.

Model	Wavelength Selection Approach	P value between Reference and Predicted Concentrations				Standard Error of Prediction Residual Error (µg/mL)			
		A	C	T	S	A	C	T	S
Global	―	0.75	0.68	0.63	0.66	4.19	4.19	1.02	1.01
	SIMPLISMA	0.77	0.86	0.53	0.77	3.78	3.78	0.98	1.10
	GA	0.63	0.68	0.99	0.35	2.78	3.02	1.16	1.29
	UVE	0.80	0.77	0.56	0.63	4.10	3.96	1.00	1.09
	MWPLSR	0.20	0.84	0.27	0.68	2.97	2.20	1.32	1.14
	SPA	0.61	0.93	0.93	0.97	3.83	3.68	1.02	1.05
Local^S^	―	0.36	0.81	0.27	0.72	1.55	1.80	0.89	0.59
Local^E^	―	0.86	0.76	0.80	0.89	3.19	4.04	0.76	0.81
Local^M^	―	0.48	0.66	0.80	0.61	2.17	1.97	0.64	0.83
Local^A^	―	0.87	0.95	0.71	0.56	2.94	2.55	0.82	0.75
Local^S^	SIMPLISMA	0.71	0.54	0.80	0.83	1.43	1.69	0.73	0.66
	UVE	0.38	0.89	0.60	0.30	1.64	1.74	1.07	0.76

SIMPLISMA = the simple-to-use interactive self-modeling mixture analysis. UVE = uninformative variables elimination. MWPLSR = moving window partial least squares. SPA = successive projections algorithm. GA = genetic algorithm. Local^S^ = local strategy based on synthetic degree of grey relation coefficient. Local^E^ = local strategy based on Euclidean distance. Local^M^ = local strategy based on Mahalanobis. Local^A^ = local strategy based on Angle. A: amaranth; C: carmine; T: tartrazine; S: sunset yellow FCF.

## Abbreviations

The following abbreviations are used in this manuscript:

PCR: Principal Components Regression
PLSR: Partial Least Squares Regression
GA: Genetic Algorithms
UVE: Uninformative Variable Elimination
MWPLS: Moving Window Partial Least Squares
SPA: Successive Projections Algorithm
S-GRC: Synthetic degree of Grey Relation Coefficient
RMSEP: Root Mean Square Error of Prediction
RMSECV: Root Mean Square Error of Prediction in Cross-Validation
LOO: Leave-One-Out
SIMPLISMA: Simple-to-use Interactive Self-modeling Mixture Analysis
GRA: Grey Relation Analysis
LOOCV: Leave-One-Out Cross-Validation
PRESS: Prediction Residual Error Sum of Squares
UV-Vis: Ultraviolet-Visible
SG: Savitzky-Golay
